# How Brunei trainee teachers cope with distress: counseling implications

**DOI:** 10.1186/s13104-017-2922-0

**Published:** 2017-11-14

**Authors:** Lawrence Mundia

**Affiliations:** Psychological Studies and Human Development Academic Group, Sultan Hassanal Bolkiah Institute of Education, Universiti Brunei Darussalam, Jalan Tungku Link, Gadong, Bandar Seri Begawan, BE 1410 Brunei Darussalam

**Keywords:** Distress, Ways of Coping, Teacher education, Trainee teachers, Brunei

## Abstract

**Objective:**

This study investigated the strategies used by student teachers when dealing with distress during training. Specifically, this study addressed the following research goals: (1) identify Ways of Coping that predict achievement on a quantitative reasoning test; (2) determine participants’ coping differences per sex, age, and ability in quantitative reasoning; and (3) reveal coping strategies that work best for each and both sexes in fostering academic achievement in quantitative reasoning. The data used in this study was from a single observation.

**Results:**

Confrontive coping, planful problem solving, and self-control were significant main effect predictors of achievement. Two separate sex-interaction variables (male with accepting responsibility and female versus accepting responsibility) were also significant predictors of achievement. Accepting responsibility was therefore helpful to both sexes in achievement. Younger participants aged 22–24 years scored significantly higher on the accepting responsibility subscale than older peers aged 25–26 years. In addition, low scorers on the quantitative reasoning test scored significantly higher on the escape avoidance coping subscale than their more-able counterparts. These findings have counseling implications for students with high support needs. A large-scale study with interview probes is recommended to learn more about this issue.

## Introduction

Coping is a natural, cautious, and defensive reaction people engage in when faced with a threat [1–3]. Several research instruments have been developed that measure a variety of coping strategies [4, 5]. The instruments currently in use differ mainly based on the target population group to be researched. Some questionnaires are for children [6], adolescents [7], or adults [8–11]. Computer-assisted online assessments of coping behaviors are also increasingly being used [8]. In addition, coping styles are distinguished in several other ways depending on whether the action taken is behavioral, emotional, cognitive, positive, negative, avoidant, or catastrophizing [1, 4, 12–15].

Students address stress in diverse ways including leisure or relaxation techniques and time management strategies to reduce anxiety levels [16, 17]. Previous research showed that men used relaxation more than women, while women used time management more effectively than men did [17]. In addition, seeking help from peers and lecturers was a common way of alleviating academic stress among students in some Asian countries such as the Philippines [18, 19], Singapore [20], and Brunei [1, 2, 15]. The ability to control emotions when faced with adversity is another way of coping with stress [21]. In Singapore and Brunei, students displayed little or no interest in consulting mental health professionals [1, 15, 20]. In China, female students were more open to receiving psychological help more so than males were [22]. Moreover, group counseling was the most popular form of intervention among secondary and tertiary students of Brunei [23].

Using the Ways of Coping instrument [10], this study probed three broad research questions: (1) what are the coping variables that are significant predictors of achievement on a quantitative reasoning test?; (2) do participants differ significantly in coping styles by sex, age, and ability in quantitative reasoning?; and (3) what interaction coping strategies work best for each and both sexes in fostering achievement in quantitative reasoning?

## Main text

### Design

This study used a field survey approach for two reasons: first, to explain verbally the purpose of the study to participants; second, to help participants who needed assistance in completing the research instruments correctly (without influencing their responses).

### Participants

This study involved a population of 80 first-year student teachers whose coping strategies had not been surveyed before. The research instruments were administered by the researcher during the academic year. Seventy-seven students voluntarily attended a psychometrics lecture that was randomly chosen for administration of the survey instruments by the researcher and instructor of the challenging psychometrics module. There was no other inclusion or exclusion criteria. The sample consisted of 59 (77%) women and 18 (23%) men (mean age = 25.53 years) (SD = 3.16).

### Instruments

Besides the demographic questionnaire, two coping scales and one test concerning psychometrics (educational testing and psychological assessment) were administered to the participants.

The Ways of Coping Questionnaire (WOC) [10] quantitative inventory was used to collect data on coping. This scale was described in detail and used in a previous Brunei study [15].

The Coping Inventory for Stressful Situations (CISS) [9] was used to address participants’ coping strategies. This instrument was also described in detail and used in two Brunei studies in the past [1, 2].

The researcher-constructed objective quantitative reasoning (QR) test, with a 4-point multiple choice response format (A, B, C, and D; one correct answer and three alternatives/distractors), was based on an introductory course in psychometrics and assessed several QR concepts (i.e., descriptive statistics, classical test theory, item analysis, correlation, reliability, validity, and standard scores). This test measured a wide range of higher-order skills (e.g., computation, interpretation, synthesis, application, evaluation, and critical thinking). Previous research used grade point average (GPA) test scores as a measure of students’ academic achievement [5, 24].

All eight WOC subscales had satisfactory reliability. Cronbach’s alpha indices ranged from 0.72 (confrontive coping) to 0.83 (seeking social support) with an average of 0.79 (distancing). Similarly, the five CISS subscales were also reliable, with alpha values ranging from 0.73 (social diversion) to 0.86 (task-oriented coping) with a mean of 0.79 (distraction). The reliability of the quantitative reasoning test was 0.84.

The criterion-related validity of the WOC and CISS scales were examined by inter-correlating the subscales within these two instruments to determine the subscales’ convergence and divergence. For example, planful problem solving coping (WOC) and task-oriented coping (CISS) correlated positively and significantly [r(77) = 0.593, p < 0.01]. This provided adequate quantitative evidence for these subscales’ convergent or concurrent validity. The reverse was also true. For instance, positive appraisal (WOC) and social diversion (CISS) were significantly negatively associated with each other [r(77) = − 0.264, p < 0.05]. This provided enough quantitative evidence for the two subscales’ divergent or discriminant validity. Similar interpretations were attached to all other inter-subscale correlations, depending on the size and direction of the obtained coefficient.

### Procedures

Permission to conduct this study was obtained from the University of Brunei Darussalam Research Ethics Committee as part of an evaluation research exercise project for the Masters of Teaching Degree program. Participants were verbally told about the purpose of this study prior to signing the consent form and completing the instruments.

### Data analysis

Research about Brunei students and none-student samples indicated that high and low scorers on psychometric and academic tests often behaved differently [1, 15, 25]. To address this concern, total scores on the QR test were dichotomized at the median value (75,000) to obtain two groups: high scorers (≥ median, coded 1, n = 39); and low scorers (< median, coded 0, n = 38). Depending on the objectives of this study and the required statistics, raw data (continuous or categorical) were then analyzed using descriptive statistics, Pearson’s correlation, t-tests for independent groups, hierarchical multiple regression analyses, and a general linear model (GLM) using SPSS Version 22. p = 0.05 (two-tailed) was chosen as the criterion value for determining significant relationships, differences, and confidence intervals (CI). In addition, tests of statistical power (e.g., effect sizes and model-fit χ^2^ indices for logistic regression analyses) were also used to determine the importance of the findings.

## Results

### WOC variables that predicted achievement on a quantitative reasoning test

The hierarchical multiple regression analysis with backward elimination produced 6 models. However, only models 1 and 6 are presented in Tables 1 and 2 for brevity. Table 1 shows the first required output from the analysis indicating the change statistics that occurred to F, R, R^2^, adjusted R^2^, SE, ΔR^2^, ΔF, and significance level for ΔF in Models 1 and 6.

**Table 1:** Change statistics from a hierarchical multiple regression of coping styles on quantitative reasoning test with backward elimination (N = 77)

Model	R	R^2^	Adj R^2^	SEest	Change statistics				
					∆R^2^	∆F	df1	df2	Sig. ∆F
1	0.970**	0.941	0.934	18.423	0.941	138.234	8	69	0.000**
6	0.969**	0.940	0.937	18.006	0.001	1.219	1	73	0.273 ns

*SEest* standard error of estimate, *ns* not significant

** p < 0.01 (two-tailed)

**Table 2:** Hierarchical multiple regression of coping styles on quantitative reasoning with backward elimination (N = 77)

Model variables	Unstandardized coefficients		Standardized coefficients	t	Sig.	95% CI for B	
	B	SEest	Beta			Lower	Upper
1							
Confrontive coping	1.706	0.967	0.209	1.765	0.082 ns	− 0.223	3.635
Distancing	− 0.057	0.739	− 0.009	− 0.077	0.939 ns	− 1.532	1.419
Self-controlling	1.550	0.719	0.306	2.157	0.034*	0.117	2.984
Seeking social support	− 0.252	0.707	− 0.041	− 0.357	0.723 ns	− 1.663	1.159
Accepting responsibility	0.237	1.214	0.027	0.195	0.846 ns	− 2.184	2.659
Escape avoidance	− 0.345	0.631	− 0.057	− 0.546	0.587 ns	− 1.603	0.914
Planful problem solving	2.140	0.769	0.385	2.784	0.007**	0.606	3.673
Positive reappraisal	0.815	0.735	0.163	1.108	0.272 ns	− 0.652	2.281
6							
Confrontive coping	1.619	0.753	0.199	2.151	0.035*	0.119	3.119
Self-controlling	1.639	0.554	0.323	2.960	0.004**	0.536	2.742
Planful problem solving	2.570	0.646	0.463	3.978	0.000**	1.283	3.858

*SEest* standard error of estimate, *ns* not significant

* p < 0.05 (two-tailed)

** p < 0.01 (two-tailed)

As indicated in Table 2, the second required output from analysis showed that the three significant predictors of achievement were: confrontive coping (B = 1.619, p = 0.035, 95% CI = 0.119–3.119), self-controlling (B = 1.639, p = 0.004, 95% CI = 0.536–2.742), and planful problem solving (B = 2.570, p = < 0.001, 95% CI = 1.283–3.85). The best predictor of achievement in quantitative reasoning was planful problem solving followed closely by self-controlling. These three WOC variables need to be incorporated in interventions for student teachers with high support needs.

### Coping style differences by sex, age, and ability in quantitative reasoning

No significant coping differences were obtained by sex on all eight dimensions of the WOC scale. However, relatively younger participants aged 22–24 years scored significantly higher on the accepting responsibility variable of WOC than did older peers aged 25–26 years [t(75) = 2.440, p < 0.01, η^2^ = 0.074]. In addition, the low scorers (n = 38) on the QR test had a significantly higher mean score on the escape-avoidance variable of WOC compared to high scorers (n = 39) [t(75) = 1.980, p < 0.05, ŋ^2^ = 0.050].

### Interaction variables that worked best with each and both sexes

Sex interactions were emphasized because of the overwhelming under-representation of men and the disproportionately high number of women in Brunei tertiary institutions [25]. According to previous research [25], men had higher support needs in quantitative subjects such as mathematics and statistics compared to women and were the most vulnerable students at risk of failing.

Using a GLM, higher-order and low-order interactions were explored; however, only four low-order interaction effects were significantly related to achievement in QR (Table 3). First, the interaction of men and accepting responsibility was a helpful joint variable with a significant positive effect in enabling struggling male participants with high support needs to succeed (B = 16.414, p = 0.035, 95% CI = 1.193–31.636, pŋ^2^ = 0.127). Second, the interaction between women and accepting responsibility also had an equally useful and helpful positive joint effect on women with high support needs (B = 13.021, p = 0.052, 95% CI = − 0.682–26.724, pŋ^2^ = 0.102). Third, the interaction of confrontive coping and planful problem solving had a significant positive and helpful effect on both sexes in facilitating academic achievement (B = 0.956, p = 0.037, 95% CI = 0.060–1.852, pŋ^2^ = 0.125). Fourth, the interaction between planful problem solving and positive reappraisal was also a significant predictor of academic achievement, but with a negative effect (B = − 0.578, p = 0.054, 95% CI = − 1.182–0.026, pŋ^2^ = 0.103).

**Table 3:** Coping strategies that were most helpful to each and both genders (N = 77)

Interaction variables	B	SE	t	Sig.	95% CI for B		Partial ŋ^2^
					Lower	Upper	
Females × confrontive coping	− 12.040	7.526	− 1.600	0.119 ns	− 27.352	3.272	0.072
Males × confrontive coping	− 10.128	7.384	− 1.372	0.179 ns	− 25.150	4.894	0.054
Females × distancing	− 3.539	5.141	− 0.688	0.496 ns	− 13.998	6.920	0.014
Males × distancing	− 1.815	5.726	− 0.317	0.753 ns	− 13.465	9.834	0.003
Females × self-controlling	7.203	4.931	1.461	0.153 ns	− 2.828	17.235	0.061
Males × self-controlling	3.895	4.744	0.821	0.418 ns	− 5.757	13.548	0.020
Females × seeking social support	− 1.252	5.539	− 0.226	0.823 ns	− 12.522	10.018	0.002
Males × seeking social support	− 3.963	6.945	− 0.571	0.572 ns	− 18.093	10.167	0.010
Females × accepting responsibility	13.021	6.735	1.933	0.052*	− 0.682	26.724	0.102
Males × accepting responsibility	16.414	7.482	2.194	0.035*	1.193	31.636	0.127
Females × escape-avoidance	− 1.584	4.505	− 0.352	0.727 ns	− 10.750	7.582	0.004
Males × escape-avoidance	− 0.215	4.494	− 0.048	0.962 ns	− 9.357	8.928	0.000
Females × planful problem solving	4.634	5.576	0.831	0.412 ns	− 6.711	15.979	0.021
Males × planful problem solving	5.378	6.414	0.839	0.408 ns	− 7.671	18.427	0.021
Females × positive reappraisal	4.255	4.222	1.008	0.321 ns	− 4.335	12.844	0.030
Males × positive reappraisal	3.898	4.676	0.834	0.411 ns	− 5.615	13.410	0.021
Confrontive coping × distancing	− 0.094	0.484	− 0.194	0.847 ns	− 1.079	0.891	0.001
Confrontive coping × self-controlling	− 0.689	0.451	− 1.528	0.136 ns	− 1.606	0.229	0.066
Confrontive coping × seeking social support	0.149	0.307	0.486	0.631 ns	− 0.476	0.775	0.007
Confrontive coping × accepting responsibility	0.617	0.684	0.903	0.373 ns	− 0.774	2.008	0.024
Confrontive coping × escape-avoidance	0.298	0.283	1.052	0.300 ns	− 0.278	0.874	0.032
Confrontive coping × planful problem solving	0.956	0.440	2.171	0.037*	0.060	1.852	0.125
Confrontive coping × positive reappraisal	0.075	0.397	0.190	0.851 ns	− 0.732	0.882	0.001
Distancing × self-controlling	− 0.213	0.217	− 0.981	0.334 ns	− 0.655	0.229	0.028
Distancing × seeking social support	− 0.330	0.376	− 0.876	0.387 ns	− 1.096	0.436	0.023
Distancing × accepting responsibility	0.194	0.533	0.364	0.718 ns	− 0.890	1.278	0.004
Distancing × escape-avoidance	0.137	0.228	0.601	0.552 ns	− 0.326	0.600	0.011
Distancing × planful problem solving	0.600	0.366	1.639	0.111 ns	− 0.145	1.344	0.075
Distancing × positive reappraisal	0.014	0.298	− 0.048	0.962 ns	− 0.622	0.593	0.000
Self-controlling × seeking social support	0.093	0.332	0.279	0.782 ns	− 0.583	0.768	0.002
Self-controlling × accepting responsibility	− 0.561	0.481	− 1.166	0.252 ns	− 1.539	0.417	0.040
Self-controlling × escape-avoidance	0.163	0.248	0.657	0.516 ns	− 0.341	0.667	0.013
Self-controlling × planful problem solving	− 0.348	0.478	− 0.727	0.472 ns	− 1.320	0.625	0.016
Self-controlling × positive reappraisal	0.579	0.378	1.532	0.135 ns	− 0.190	1.347	0.066
Seeking social support × accepting responsibility	− 0.097	0.376	− 0.257	0.799 ns	− 0.863	0.669	0.002
Seeking social support × escape-avoidance	0.136	0.271	0.502	0.619 ns	− 0.415	0.686	0.008
Seeking social support × planful problem solving	− 0.111	0.312	− 0.354	0.726 ns	− 0.746	0.525	0.004
Seeking social support × positive reappraisal	0.197	0.319	0.618	0.541 ns	− 0.451	0.845	0.011
Accepting responsibility × escape-avoidance	0.041	0.433	0.095	0.925 ns	− 0.840	0.922	0.000
Accepting responsibility × planful problem solving	− 0.411	0.577	0.713	0.481 ns	− 1.585	0.763	0.015
Accepting responsibility × positive reappraisal	− 0.466	0.425	− 1.096	0.281 ns	− 1.332	0.399	0.035
Escape-avoidance × planful problem solving	− 0.112	0.311	− 0.360	0.721 ns	− 0.744	0.520	0.004
Escape-avoidance × positive reappraisal	− 0.455	0.297	− 1.531	0.135 ns	− 1.059	0.150	0.066
Planful problem solving × positive reappraisal	− 0.578	0.297	− 1.947	0.054*	− 1.182	0.026	0.103

*ns* not significant

* p < 0.05 (two-tailed)

## Discussion

The fact that many WOC and CISS subscales were positively and significantly related was no surprise as this concurred with the reviewed literature [9, 10]. However, an interesting finding was the significant interaction effect between confrontive coping and planful problem solving that was helpful to both sexes regarding achievement. Since planful problem solving was related with task-oriented coping in, this implied that it operated like task-oriented coping, as suggested by previous literature [9]. This observation was consistent with and supportive of the findings in a recent, related study [1] about the value and importance of task-oriented coping in Brunei student teacher samples. Task-oriented coping worked like productive coping in the Adolescent Coping Scale [7] and in a similar way to active coping in the COPE Scale [8]. These forms of coping are also known as behavioral engagement coping [4, 13, 14] or as proactive coping [11]. According to the literature, productive coping is correlated with academic achievement and can help diverse categories of students to succeed [5].

Accepting responsibility in the WOC questionnaire [10] was positively and significantly related to the emotion-oriented coping subscale of the CISS [9]. The ability to deal appropriately and effectively with emotional aspects of distress often enables people to successfully resolve their problems [21]. Escape avoidance was another WOC variable that was related to emotion-oriented coping. This suggests that participants who scored low on the QR course/test, but scored high on the escape avoidance variable, could perform well if they knew how to control and use their emotions wisely.

Research argues that coping strategies are alterable and teachable [4, 13, 14, 21]. Group counseling was the type of intervention that appealed to Brunei students [23]. Based on the findings, it is recommended that motivational talks, seminars, and workshops be conducted to provide group training to distressed Masters of teaching students on the effective use of coping strategies related to academic achievement. In addition, research has indicated that some Asian students scored high on dysfunctional coping strategies [18–20].

### Limitations

The two major limitations of the present study included the following:

- Lack of a qualitative section with interview probes to supplement self-report quantitative survey data; and
- Use of a non-diverse and small-to-moderate sample size (N = 77) inhibited the generalization of the findings to other student teacher groups and necessitated recommending a large-scale mixed methods study.

## Abbreviations

UBD: University of Brunei Darussalam
SHBIE: Sultan Hassanal Bolkiah Institute of Education
MTeach: Master of Teaching
WOC: Ways of Coping
CISS: Coping Inventory for Stressful Situations
SPSS: Statistical Package for Social Sciences
GLM: general linear model
CI: confidence interval
